# Integrin αvβ8 serves as a Novel Marker of Poor Prognosis in Colon Carcinoma and Regulates Cell Invasiveness through the Activation of TGF-β1

**DOI:** 10.7150/jca.43826

**Published:** 2020-04-06

**Authors:** Mingliang Zhou, Jun Niu, Jinshen Wang, Huijie Gao, Muhammad Shahbaz, Zhengchuan Niu, Zequn Li, Xueqing Zou, Benjia Liang

**Affiliations:** 1 Department of Gastrointestinal Surgery, Shandong Provincial Hospital Affiliated to Shandong First Medical University, Jinan 250021, Shandong, China; 2 Department of Gastrointestinal Surgery, Shandong Provincial Hospital Affiliated to Shandong University, Jinan 250021, Shandong, China; 3 Department of Hepatobiliary Surgery, Qilu Hospital, Shandong University, Jinan 250012 Shandong, China; 4 Department of General Surgery, Zhongshan Hospital, Fudan University, Shanghai 20032, China; 5 Department of Gastrointestinal Surgery, Affiliated Hospital of Qingdao University, Qingdao 266003, Shandong, China

**Keywords:** integrin αvβ8, colon cancer, prognostic factors, cell invasiveness, TGF-β1

## Abstract

Integrin αvβ8 expressed on tumor cells executes crucial regulatory functions during cell adhesion in the tumor microenvironment and supports the activation of TGF-β1. This study aimed to investigate the expression of integrin αvβ8 and its clinical significance in colon cancer, in addition to its influence on the invasion and migration of cancer cells. Our results showed that integrin αvβ8 was an indicator of progression and poor prognosis in patients with colon cancer. Moreover, integrin αvβ8 significantly promoted the invasion and migration of colon cancer cells by the activation of TGF-β1 and upregulation of metalloproteinase-9. Furthermore, suppression of integrin αvβ8 was found to inhibit the growth of colon cancer *in vivo*. Our results indicate that integrin αvβ8 promotes tumor invasiveness and the migration of colon cancer through TGF-β1 activation and is a potential prognostic biomarker. This study may provide clues to further understand the manner in which the tumor microenvironment mediates the development of colon cancer and develop strategies for novel therapeutic targets in the prevention and treatment of colon cancer.

## Introduction

Colon cancer is the third most common cancer and the fourth leading cause of cancer-related death worldwide 1. Recent developments in treatment modalities, including radical resection-based surgery, and new chemotherapy regimens have achieved a marked improvement in the short-term survival of patients with colon cancer. Nevertheless, long-term prognosis in advanced cases remains unsatisfactory because of distant metastases 2, 3. Consequently, there is an urgent need to further understand the potential molecular mechanism involved in tumor progression and migration of colon cancer as well as to explore new and effective predictors of patient outcomes and novel therapeutic approaches to colon cancer.

Integrins are heterodimeric transmembrane cell surface receptors consisting of one α- and one β-subunit. At least 24 distinct integrin heterodimers are formed by different combinations of 18 α-subunits and 8 β-subunits 4, 5. They execute crucial regulatory functions during cell adhesion to the extracellular matrix (ECM) and immunoglobulin superfamily molecules 6. Moreover, integrins are capable of bidirectional signaling across cell membranes, referred to as “outside-in” and “inside-out” signaling, which results in information exchange between the ECM proteins and intracellular molecules 4, 7. Among the 24 human integrin subtypes known to date, 8 integrin dimers (αvβ1, αvβ3, αvβ5, αvβ6, αvβ8, α5β1, α8β1, and αIIbβ3) recognize RGD (arginine-glycine-aspartic acid) peptide motifs in ECM proteins. These integrins constitute a receptor subfamily instrumental in cancer progression and metastasis 8-12.

Integrin αvβ8 is a transmembrane glycoprotein that exclusively heterodimerizes with the αv subunit 13. Integrin αvβ8 can bind to numerous ECM proteins and is reported to be a primary receptor for latent TGF-β, which is an inactive complex produced by kinds of cells. Integrin αvβ8 binding to the RGD motif contained in the LAP of latent TGF-β complexes mediates TGF-β activation and receptor pathway signaling 14, 15. Most of the proliferation, migration, and immune effects relevant to malignant tumors have been attributed to integrin-activated TGF-β^16^, ^17^. In contrast to other integrins, αvβ8 activates latent TGF-β in a protease-dependent manner by the co-localization of membrane-bound protease MT1-MMP/MMP14. Additionally, αvβ8 binds to LAP and brings the latent complexes in proximity with the membrane-bound protease that cleaves LAP 18. αvβ8 has been detected in tumor cells of various carcinomas, including lung, ovarian, endometrial, melanoma, breast, prostate, colon, skin, and stomach. αvβ8 expression in human cancer cells is involved in the progression of epithelial malignancies; increased αvβ8 expression is also associated with decreased survival in non-small cell lung carcinoma, triple-negative basal-type breast cancer, and advanced ovarian cancer^19^-^21^. Additionally, αvβ8-mediated TGF-β activation regulates tumor immune tolerance, which results in the decreased infiltration of cytotoxic T cells and proinflammatory tumor-associated macrophages to the tumor center 19, 22, 23.

Integrin αvβ8 is far less studied than other members of the αv integrin subfamily; however, it is not only structurally but also functionally related to integrin αvβ6 24. Our previous research showed that integrin αvβ6 plays an important role in the invasiveness, metastasis, and degradation of the ECM of colorectal cancer and demonstrated that there is a direct link between extracellular signal-regulated kinase-2 (ERK2) and β6, which is essential for β6-mediated ERK2 activation and corresponding downstream effects^9^, ^25^-^27^. Nevertheless, the role of integrin αvβ8 in the progression, invasion, and migration of colon cancer is still largely unknown and its precise mechanism remains to be clarified. In the present study, we detected the expression of integrin αvβ8 in colon cancer and investigated its tumor prognostic significance. Moreover, we explored the potential role and underlying mechanism of αvβ8-mediated TGF-β activation in cell migration in colon cancer.

## Materials and Methods

### Clinical samples and colon cancer cell lines

Patients underwent successful tumor resection surgery at the Department of Gastrointestinal Surgery, Shandong Provincial Hospital, Jinan, Shandong, China, between June 2012 and July 2016. There were 90 males and 75 females with a median age of 64.6 years and an age range of 42-81 years. To be included, patients with colon cancer must have received surgical resection as the initial treatment modality without major perioperative complications, as confirmed by histological examination. Colon cancer was classified into right hemi colon cancer (including cecum, ascending colon, and right transverse colon) and left hemi colon cancer (including left transverse colon and descending colon) based on anatomical location of tumors. The pathologic tumor-node-metastasis (TNM) classification of the International Union Against Cancer (2009). The study complied with the requirements of The Ethics Committee of Shandong Provincial Hospital, Jinan, Shandong, China.

Caco-2, RKO, LoVo, SW620, SW480, DLD-1, HT-29, and HCT-116 colon cancer cell lines were purchased from American Type Culture Collection. All cells were grown in Dulbecco's Modified Eagle's Media supplemented with 10% fetal bovine serum and antibiotics.

### Immunohistochemistry

Representative areas of the tumor were selected based on hematoxylin-eosin (HE) staining. Tissue sections were incubated for 60 min at 65°C and rehydrated by using xylene and ethanol series. The tissues were then dipped thrice in phosphate buffered saline (PBS). Then, microwave antigen retrieval was performed by the following method: sections were spaced in antigen retrieval buffer (pH 6.4) for microwaving, with high temperature for 5 min and 40°C for 15 min. After cooling to room temperature and washing in PBS, the tissues were quenched of endogenous peroxidase by 3% H_2_O_2_ for 20 min and blocked with goat serum at 37°C for 30 min, followed by incubation with anti-β8 antibodies (ab80673, 1:100, Abcam, US) or anti-CD8 antibodies (sc-1177, 1:100, Santa Cruz Biotechnology, US) overnight at 4°C. On the following day, tissues were incubated with the universal IgG antibody-Fab-HRP polymer for 30 min. Subsequently, diaminobenzidine and hematoxylin were stained and terminated sequentially. Normal mouse IgG was substituted for the primary antibody as the negative control. Finally, the samples were observed under a light microscope (Olympus Corp, Tokyo, Japan).

### Evaluation of immunohistochemistry

Integrin αvβ8 was expressed both inside the cells and on the cellular membrane, mainly seen on the internal surface of the tumor cell membrane. Expression levels were evaluated by three individuals based on the average intensity and percentage of positively stained cells. The intensity of staining was scored as 0 (no staining), 1 (weak staining = light yellow), 2 (moderate staining = yellow brown), and 3 (strong staining = brown). The percentage of stained cells was scored as 0 (no positive cells), 1 (less than 25% positive cells), 2 (25-50% positive cells), 3 (more than 50-75% positive cells), and 4 (more than 75% positive cells). Scoring was validated by a consulting histopathologist. Positive staining was judged by the presence of an unequivocal brown staining in ≥10% of tumor cells.

Tumor-infiltrating lymphocytes (TILs) were evaluated according to previous reports. Briefly, tumor-infiltrating CD8^+^T lymphocytes were counted separately by intraepithelial or stromal localization. CD8^+^T lymphocytes that infiltrated into cancer cell nests were designated as intraepithelial CD8^+^T lymphocytes. Three areas of intraepithelial CD8^+^T lymphocytes with the most abundant infiltration were selected, and the average count was calculated.

### Western blot analysis

Cells were harvested and lysed. Samples with equal amounts of protein were loaded onto an SDS-PAGE (sodium dodecyl sulfate-polyacrylamide gel electrophoresis) gel and electrophoresed. Subsequently, the separated proteins were transferred onto polyvinylidene fluoride membranes. The membranes were probed with primary antibodies overnight at 4°C, followed by horseradish peroxidase-labeled secondary antibodies. Immunoreactive bands were visualized using the electrochemiluminescence method, and optical density was analyzed with ImageJ. Values were expressed as a fold of GAPDH.

### Quantitative real-time PCR

Total RNA was extracted from cells by Trizol (Invitrogen). The reverse transcription reaction was performed using RevertAid First Strand cDNA Synthesis Kit (Fermentas) according to manufacturer instructions. cDNA obtained from the reverse transcription reaction was analyzed by a real-time PCR thermocycler. Quantitative values were obtained by the threshold cycle value. Relative mean fold change in expression ratios was calculated by the 2^-ΔΔCT^ method. The primers for human integrin β8 were as follows: 5′-ACCAGGAGAAGTGTCTATCCAG-3′ (forward) and 5′-CCAAGACGAAAGTCACGGGA-3′ (reverse). The housekeeping gene GAPDH served as internal control.

### Cell adhesion assay and active TGF-β reporter cell assay

Untreated polystyrene 96-well flat plates were coated with LAP (R&D Systems, Minneapolis, MN, USA) at 37°C for 1 h. As a negative control, wells were coated with 1% bovine serum albumin. Wells were washed with PBS. Cells (5 × 10^4^ cells/well) were harvested and added to each well. Cells were centrifuged at 10G for 3 min to ensure uniform settling of cells and incubated for 1 h at 37°C. Non-adherent cells were then removed by centrifugation (top-side down) at 10G for 5 min. The attached cells were fixed and stained with 1% formaldehyde/0.5% crystal violet/20% methanol for 30 min at room temperature. After washing with PBS, adherence was determined by absorption at 595 nm on a microplate reader.

To determine the TGF-β activation, transformed mink lung epithelial cells (TMLC) stably transfected with a portion of the plasminogen activated inhibitor-1 promoter were cocultured with test cells as described previously 28. TMLC cells are highly responsive to TGF-β and produce a very low background of TGF-β activation. TMLC cells can thus be used in coculture with other cell lines to test for the presence of active TGF-β using luminescence as a readout. Colon cancer cells (1.5 × 10^4^ cells/well) were co-cultured with TMLCs (1.5 × 10^4^ cells/well) in a 96-well plate overnight at 37°C. Then, luciferase levels were measured using the Luciferase assay system kit according to manufacturer instructions (Promega, Madison, WI, USA). An active TGF-β standard curve was used to calculate levels of active TGF-β from luminescence intensity.

### Cell invasion assays

The invasive ability of colon cancer cells was assessed using a 24-well transwell chamber (cell invasion assay kit), by calculating the number of cells that passed through a polycarbonate membrane (Corning Costar). The polycarbonate surface of each chamber was covered with 20 μl matrigel (BD Biosciences; 1:4 dilution). Cells were pretreated with β8-siRNA or β8 antibodies (E-6, Santa Cruz, US) overnight. Cells (1 × 10^5^ cell /well) in 150 μl serum free medium with latent TGF-β1 (2 μg/ml) were seeded into the upper chamber. After culture for 24 h at 37°C, the upper surface of the Transwell membrane was wiped gently with a cotton swab to remove non-migrating cells. The membranes were fixed with methanol and stained with 0.1% crystal violet. The number of invaded cells in five random optical fields of each filter from triplicate inserts was averaged. Migration index was plotted as the mean ± standard deviation (SD) against the dose with control cells set to 100%. Shown are the means ± SD of three independent experiments.

### Wound-healing assays

The monolayer wound-healing assay was used to assess cell migration. In total, 5 × 10^5^ cells were seeded in 6-well plates, incubated overnight and, then, pretreated with β8-siRNA or β8 antibodies. After achieving 90% confluency, cells were pretreated with latent TGF-β1 (2 μg/ml) and scratched with a sterile pipette tip; the floating cells were removed with PBS, and the cells were cultured for 24 h. Photographs were taken at 0 and 24 h along the scrape line using a microscope. The results are expressed as the actual wound closure distance.

### Enzyme-linked immunosorbent assay (ELISA)

Cells (1 × 10^5^ cells/well) were cultured in a 6-well cell culture plate and treated for 24 h with β8-siRNA or β8 antibodies. Then, cells were incubated in serum free medium with latent TGF-β1 (2 μg/ml) overnight. The culture supernatant was collected; the level of secreted matrix metalloproteinase-9 (MMP-9) was analyzed via enzyme-linked immunosorbent assay (ELISA) following manufacturer guidelines (R&D). Samples were assayed in triplicate and calibrated against a standard curve.

### Gelatin zymography

Cells (1 × 10^5^ cells/well) were cultured in a 6-well cell culture plate and treated for 24 h with β8-siRNA or β8 antibodies. Then, cells were incubated in serum free medium with latent TGF-β1 (2 μg/ml) overnight. Subsequently, MMP-9 activity was determined by gelatin zymography, using a Gelatin Zymo Electrophoresis Kit (Genmed Scientifics, Arlington, MA, USA), according to manufacturer instructions. The conditioned media of cells incubated in serum-free medium for 48 h was removed and centrifuged. SDS-PAGE were copolymerized with gelatin. After electrophoresis, the gels were renatured in 2.5% Triton X-100 and incubated at 37°C for 24 h in 5 mM calcium chloride and 50 mM Tris-hydrogen chloride buffer (pH 7.5), containing 0.05% sodium azide. The gels were stained with 0.5% Coomassie blue R-250 and destained in 10% methanol and 5% acetic acid in water. Gelatinolytic activities were detected as transparent bands on a blue background. Band intensities were quantified using ImageJ.

### Tumorigenicity assay

The ability of human colon cancer cells to form tumors in BALB/C female nude mice was assessed by measuring length and breadth of tumors following subcutaneous inoculation of 10^6^ viable tumor cells suspended in 100 μl of standard complete culture medium (Dulbecco's Modified Eagle's Medium). Each group had 8 mice. The growth of primary tumors was monitored by measuring tumor diameters with electronic calipers every other day continuously from the second week after injection. Volumes were calculated using the formula (length)×(width)^2^/2. After 3 weeks, the mice were sacrificed and the tumors were dislodged and weighed. Experiment was repeated three times. For the determination of Ki-67 expression, paraffin-embedded tumor tissues were immunostaining with primary Ki-67 antibodies. For assessment of Ki-67 index, over 1000 tumor cells per specimen were counted on three photographs. The Ki-67 index was estimated by the percentage of Ki-67-positive cancer cells in all of the counted tumor cells. All animal studies were conducted using a protocol approved by the institutional animal care and use committee at Shandong Provincial Hospital Affiliated to Shandong University.

### Statistical analysis

A chi-square test of cross-tabulations and a Fisher exact test were used to examine the relationship between the expression of integrin αvβ6 and the clinicopathologic characteristics of patients. Survival analyses were conducted by the Kaplan- Meier method and the log-rank test. Both univariate and multivariate analyses for cancer specific deaths were done with the Cox proportional hazard model. Data are presented as the mean ± SD; all measurements were obtained from at least three independent experiments. P <0.05 was considered statistically significant. Statistical analyses were performed using GraphPad Prism (GraphPad Software, Inc.).

## Results

### Analysis of integrin αvβ8 expression in human colon cancer and its relationship with clinicopathological factors of colon cancer

**Figure 1:**
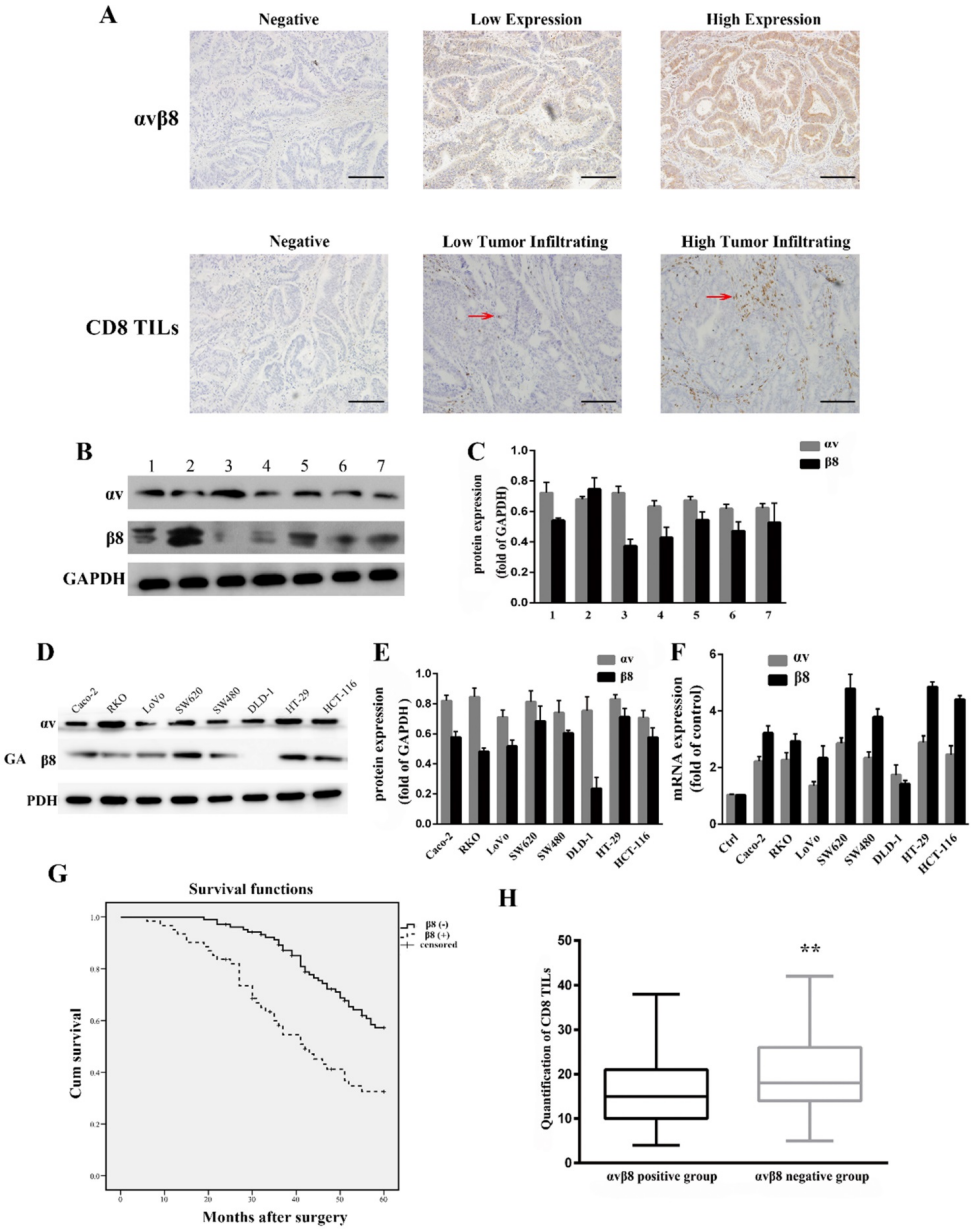
** Expression of integrin αvβ8 in colon cancer. The relationship between integrin αvβ8 and overall survival and CD8+TILs. A.** Immunohistochemical expression of integrin αvβ8 and CD8^+^TILs in colon cancer tissues. Bar = 100 μm. Red arrows indicate tumor infiltrating CD8^+^ cells **B.** Expression of the αv and β8 subunit in seven different colon cancer tissues as analyzed by western blot. All expressed similar levels of αv integrin protein, and six samples expressed robust levels of β8 subunit, with one sample expressing lower level of β8 subunit. **C.** Quantification of αv and β8 subunit expression in three independent experiments performed. Values are expressed as a fold of GAPDH. Data represent the mean ± SD (n = 3). **D.** Expression of αv and β8 subunit in different colon cancer cells as analyzed by western blot. All cell lines expressed similar levels of αv integrin protein. SW480, SW620, HT-29, HCT-116, RKO, and Caco-2 cells expressed high level of β8 integrin. **E.** Quantification of αv and β8 subunit expression measured by Western blot in three independent experiments. Values are expressed as a fold of GAPDH. Data represent the mean ± SD (n = 3). **F.** Expression of αv and β8 mRNA determined by real-time PCR in three independent experiments. **G.** Association between integrin αvβ8 expression and overall survival of colon cancer patients. **H.** Box-and-whisker graph of CD8^+^TILs. ^**^P < 0.01.

Integrin αvβ8 expression in colon cancer was determined by immunohistochemistry. For 165 primary colon cancer samples, αvβ8 staining was observed both in the membrane and cytoplasm of tumor cells. Positive αvβ8 expression was observed in 61 patients (36.9%; Figure 1A, Table 1). Integrin αvβ8 was stained in colon cancer tissues but negligibly in para-neoplastic normal tissues. We also compared integrin expression levels by immunoblots prepared from seven different freshly resected colon cancer samples. All samples expressed similar levels of αv integrin protein; six of the seven samples expressed robust levels of β8 integrin protein, with one sample expressing lower levels of β8 integrin (Figure 1B and C).

We used anti-αv integrin and anti-β8 integrin antibodies to detect integrin protein expression levels in eight different human colon cancer cell lines. All cell lines expressed similar levels of integrin αv, whereas integrin β8 was expressed at varying levels in different cell lines (Figure 1D and E). The human colon cancer cell lines SW480, SW620, HT-29, HCT-116, RKO, and Caco-2 expressed high levels of integrin β8. In contrast, DLD-1 cells had decreased expression of integrin β8. Real-time PCR was used to analyze the expression of integrin αvβ8 at the mRNA level. SW620 and HT-29 expressed higher levels of integrin αvβ8 mRNA, whereas HCT-116 and SW480 had moderate αvβ8 mRNA expression (Figure 1F).

We investigated the relationship between integrin αvβ8 expression and clinicopathological factors of colon cancer from the 165 primary samples. There was a substantial association between integrin αvβ8 expression and N stage, M stage, TNM stage, and tumor cell differentiation of colon cancer. The percentage of late N (N1, N2) and M stage (M1) among integrin αvβ8-positive samples was much higher than these among integrin αvβ8-negative samples. In other words, integrin αvβ8-negative samples had an increased percentage of early N and M stage. Likewise, among integrin αvβ8 positive samples, the percentage of TNM III-IV-stage was higher than that of TNM I-II-stage. Integrin αvβ8-positive specimens revealed a higher percentage of poor differentiation than αvβ8-negative specimens (Table 1). Moreover, patients with αvβ8 expression (median survival time = 43 months) had an obviously poorer overall survival rate than those with negative αvβ8 expression (median survival time = 56 months; P<0.001 log-rank test: χ2 = 12.435; Figure 1G).

### Correlation between integrin αvβ8 expression and tumor-infiltrating CD8^+^T lymphocytes in colon cancer

Integrin αvβ8 detected on tumor cells serves as a platform for TGF-β1 activation on tumor-infiltrating immune cells, which has been suggested to be a dominant mechanism of tumor immunosuppression 22. Tumor-infiltrating CD8^+^T lymphocytes, which are a crucial component of the cellular immune system, constitutes the effector arm of adaptive antitumor immunity^29^, ^30^. We determined the association between integrin αvβ8 expression and CD8^+^TILs in colon cancer by immunohistochemistry. Among 165 colon cancer cases, a low CD8^+^TIL density was detected in 73 (44.2%) cases, whereas a high CD8^+^TIL density was present in 92 (55.7%) cases. There was significant correlation between integrin αvβ8 expression and CD8^+^TIL density (P<0.001). Among the low CD8^+^TIL density cases, positive αvβ8 expression was detected in 52.0% of cases (Table 1). Moreover, there was a significant inverse correlation between CD8^+^TIL count and αvβ8 expression (Figure 1H). Collectively, it is likely that integrin αvβ8 expression on tumor cells prohibits the invasion of CD8^+^TILs, resulting in immunosuppression of the tumor.

### Univariate and multivariate analysis for prognosis of patients with colon cancer

To determine the prognostic value of integrin αvβ8 expression, univariate and multivariate analysis was performed using the Cox proportional hazards regression model. In addition to age at diagnosis, M stage, and poor differentiation, integrin αvβ8 expression was able to predict a poor prognosis in univariate analysis (P < 0.05; Table 2). Variables deemed to be significant were used to conduct multivariate analysis. The results revealed that αvβ8 expression was an independent unfavorable prognostic factors (relative risk: 1.681; P = 0.031). Moreover, age at diagnosis and M stage were also independent prognostic factors (relative risk: 1.085 and 1.854; P =0.007 and 0.037, respectively; Table 2).

### Integrin αvβ8 in colon cancer cells promotes TGF-β1 activation through adhesion to LAP

**Figure 2:**
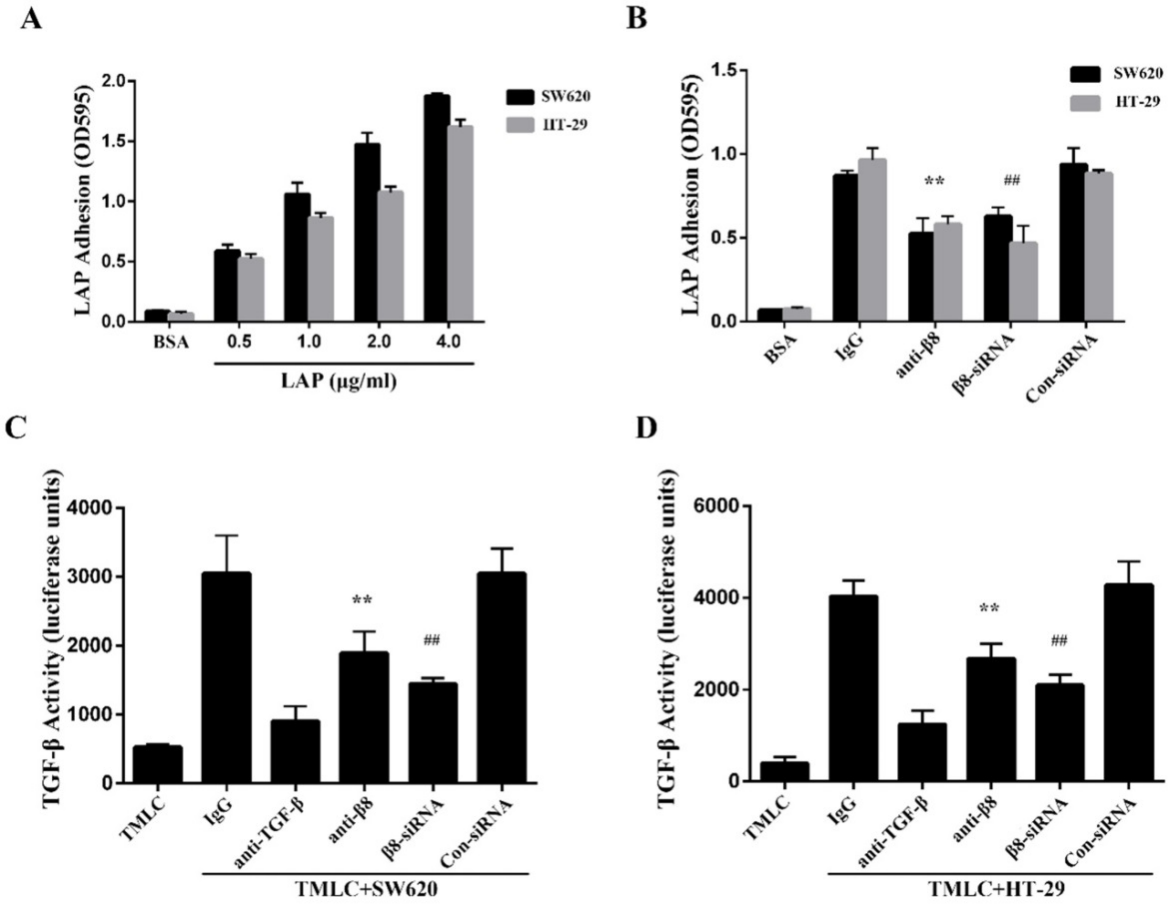
** Integrin αvβ8 mediates colon cancer cells adhesion to LAP and regulates activation of TGF-β. A.** Integrin αvβ8 binding to LAP in SW620 and HT-29 colon cancer cells as detected by adhesion assays. Integrin αvβ8 binding to LAP was elevated as LAP concentrations increased. BSA was used as negative control. **B.** Integrin αvβ8 binding to LAP in SW620 and HT-29 colon cancer cells treated with β8-antibodies or β8-siRNA. β8-Antibody or β8-siRNA inhibited cell adhesion to LAP. **C and D.** Co-cultured with transformed mink lung epithelial cells (TMLC) to detect the TGF-β activity. The activity of TGF-β was abolished when cells were treated with β8-antibody or β8-siRNA. ^**^P < 0.01 versus IgG; ^##^P < 0.01 versus con-siRNA. Results are representative of three independent experiments.

To determine whether integrin αvβ8 binds to LAP in colon cancer cells, we performed adhesion assays with the SW620 and HT-29 colon cancer cell lines. We found both cell lines were able to adhere to LAP; adhesion increased in tandem with the concentration of LAP (Figure 2A). Adhesion of the SW620 and HT-29 cells to LAP was abolished by β8 integrin antibodies. Moreover, when the expression of integrin αvβ8 on SW620 and HT-29 cells was inhibited by β8-siRNA, cell adhesion to LAP was also reduced (Figure 2B).

Integrin αvβ8 is a receptor for ECM-bound latent TGF-β1 and mediates its activation and subsequent receptor engagement 31. To investigate roles for αvβ8-mediated TGF-β1 activation in colon cancer cells, we co-cultured SW620 and HT-29 cells with TMLC. Both cell lines caused a significant increase in luciferase levels when compared to TMLC alone. This increase was abolished by either the β8 integrin antibody or anti-TGF-β1 antibody (Figure 2C and D). Additionally, SW620 and HT-29 transfected with con-siRNA or β8-siRNA were co-cultured with TMLC. Luciferase activity decreased nearly 2-fold in cells transfected with β8-siRNA. These results suggest that integrin αvβ8 mediates latent TGF-β1 activation within colon carcinomas by interacting with LAP.

### Integrin αvβ8-mediated TGF-β1 activation and signaling are essential for invasion and migration of colon cancer

**Figure 3:**
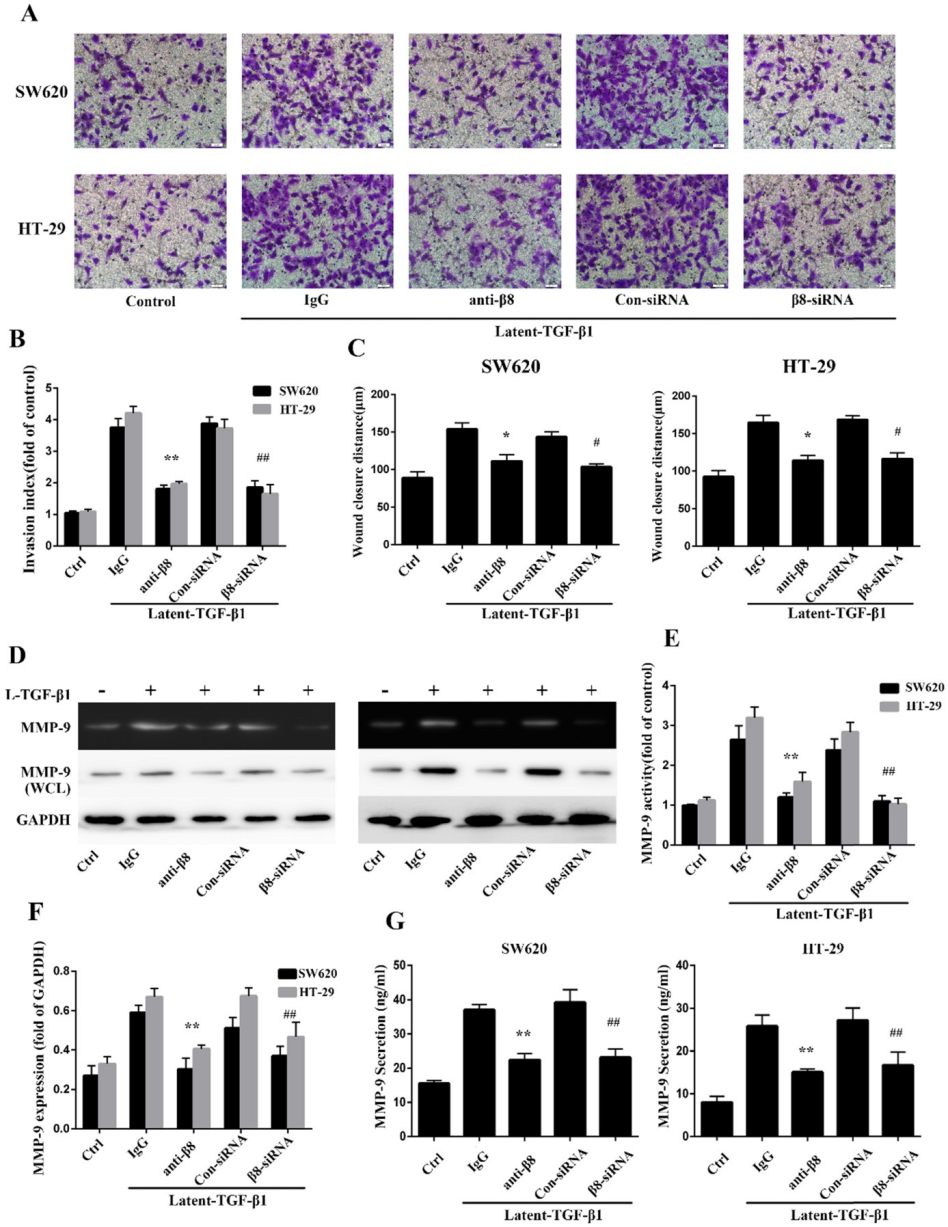
** Integrin αvβ8 promotes migration and invasion of colon cancer cells and upregulates MMP-9. A.** Cell invasion was measured with the Transwell experiment. TGF-β1 was found to increase the cell invasion of colon cancer cells; this increase could be inhibited by β8-antibody or β8-siRNA. **B.** Invasion index was calculated by three independent experiments. **C.** Cell migration was examined by wound healing assay. Cell migration was also inhibited by β8-antibody or β8-siRNA for both cell lines. **D.** MMP-9 activity and expression under the condition of latent TGF-β1 was detected, respectively, by gelatin zymography and western blot. β8-Antibody or β8-siRNA was able to reduce both activity and expression of MMP-9. **E.** Quantification of MMP-9 activity. **F.** Quantification of MMP-9 expression. **G.** Secretion of MMP-9 in SW620 and HT-29 colon cancer cells as examined by enzyme-linked immunosorbent assay (ELISA). Shown are mean±SD of three independent experiments.^ **^P < 0.01, ^*^P < 0.05 versus IgG; ^##^P < 0.01, ^#^P < 0.05 versus con-siRNA.

TGF-β1 have been reported to promote migration and invasion of cancers^31^, ^32^. To further explore whether integrin αvβ8 is required for TGF-β1-mediated cell migration of colon cancer, we examined SW620 and HT-29 cell invasion under the condition of latent TGF-β1. Cell invasion assays showed that latent TGF-β1 treatment dramatically increased cell invasion for both cell lines. Then specific αvβ8 blocking antibody and specific β8 siRNA were used. Compared with that of the control cells, inhibition of αvβ8 by antibody or siRNA significantly decreased latent TGF-β1-induced cell invasion of SW620 and HT-29 cells (Figure 3A and B).

Cell migration was next analyzed by *in vitro* wound-healing assays. The addition of latent TGF-β1 enhanced directional migration in SW620 and HT-29 colon cancer cells, compared with untreated cells (Figure 3C). Similarly, we found that αvβ8 antibodies or β8-siRNA significantly inhibited wound healing under the condition of latent TGF-β1 (Figure 3C). These data suggest that TGF-β1 might promote cell migration and invasion of colon cancer via integrin αvβ8.

### Integrin αvβ8 mediates regulation of MMP-9 by TGF-β1 activation in colon cancer cells

It has been reported that TGF-β1 enhances tumor invasion by stimulating MMPs, such as MMP-9 33-35. To determine whether integrin αvβ8 could induce the stimulation of MMP-9 by activating TGF- β1 in colon cancer cells, the activity of MMP-9 was examined by zymography on SW620 and HT-29 cell lines with the treatment of latent TGF-β1. For integrin αvβ8 positive cell lines, latent TGF-β1 promoted the activity of MMP-9. However, this upregulation could be inhibited by prior incubation of cell lines with αvβ8 antibodies or β8-siRNA (Figure 3D and E). The expression of MMP-9 in whole-cell lysates of colon cancer cells was also determined by immunoblotting. It was observed that latent TGF-β1 could increase the expression of MMP-9 (Figure 3D and F). This increase was inhibited by αvβ8 antibodies or β8-siRNA. Moreover, we examined the levels of secreted MMP-9 in the cell culture media. Similarly, the secretion of MMP-9 could be enhanced by latent TGF-β1, which was abolished by αvβ8 antibodies or β8-siRNA (Figure 3G). Thus, integrin αvβ8 was required for upregulation of MMP-9 by TGF-β1 signaling.

### Silencing of integrin αvβ8 expression inhibits tumor growth of colon cancer *in vivo*

**Figure 4:**
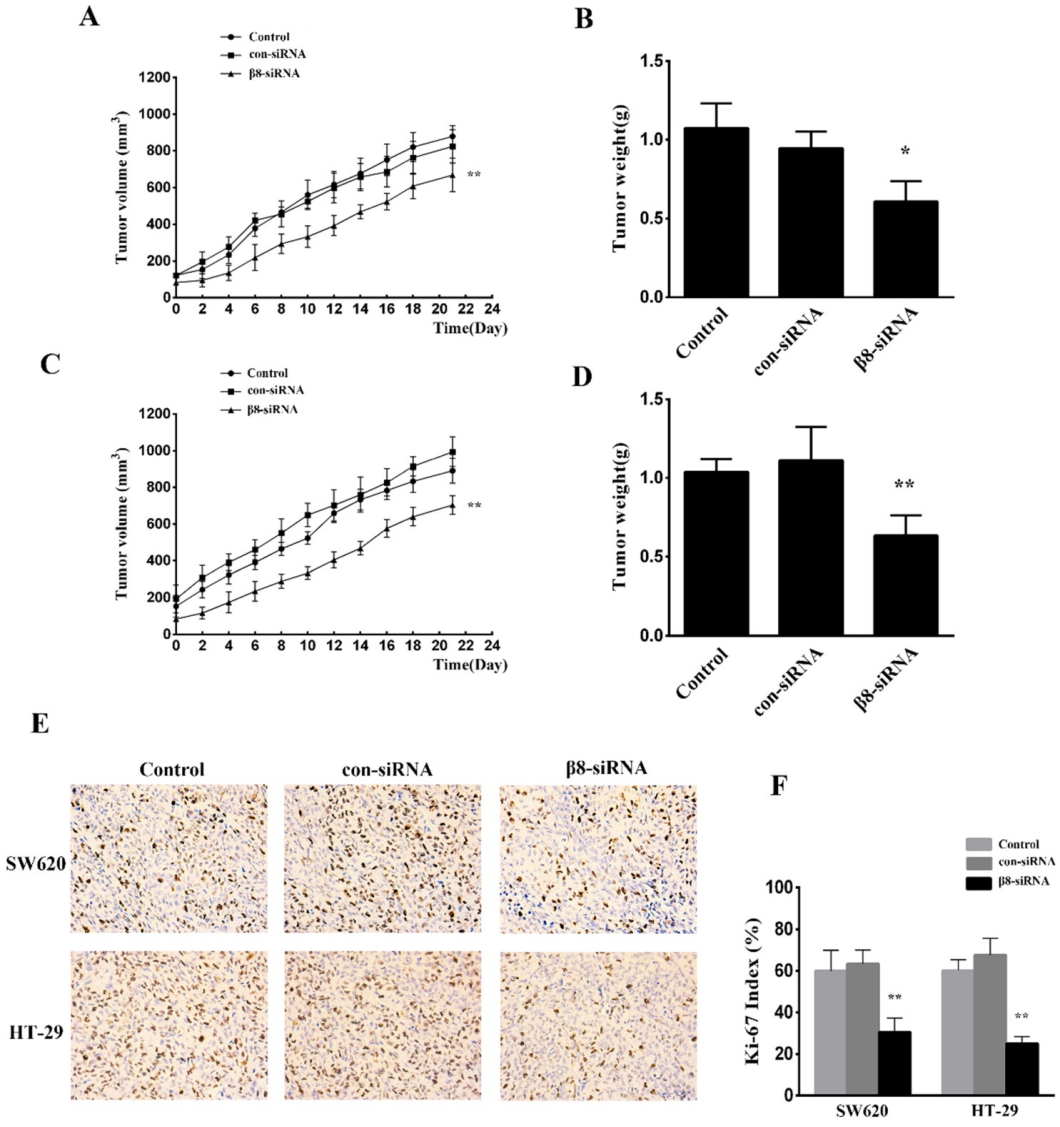
** Knocking down integrin αvβ8 expression reduces the growth of colon cancer tumor xenografts. A.** The growth curve of tumors for SW620 colon tumor xenograft models. **B.** The mean tumor weight of SW620 colon tumor xenograft. n= 8 in each group, ^**^P < 0.01, ^*^P < 0.05 versus con-siRNA. **C. D.** The growth curve and mean tumor weight of HT-29 colon tumor xenograft. **E.** Immunohistochemical expression of Ki-67 in the tissue of colon tumor xenograft. **F.** Ki-67 index is shown. Shown are mean±SD of three independent experiments.^ **^P < 0.01 versus con-siRNA.

To examine the effect of αvβ8 suppression on* in vivo* tumor growth, SW620 and HT-29 colon cancer cells transfected with β8-siRNA or con-siRNA were inoculated into BALB/C female nude mice. Suppression of αvβ8 greatly delayed xenograft growth for both colon cancer models (Figure 4A and C). The weight of isolated tumors from the β8-siRNA group were significantly reduced when compared to control (Figure 4B and D). Additionally, the tumor growth was detected by immunohistochemical analysis of Ki-67 staining. Silencing of integrin αvβ8 significantly suppressed the expression of Ki-67 in tumor tissues and reduced the Ki-67 proliferation index by about 30% compared to control groups (Figure 4E and F).

## Discussion

Cellular recognition relies on cell-ECM or cell-cell communication which is indispensable for individual tumor cells in the microenvironment and is required in all solid tumors 36. Integrins are performing bidirectional signaling through cellular membranes, which results in “messages” exchange between the ECM and cells or between individual cell 37. Many integrins are highly expressed in carcinomas of the colon, stomach, breast and pancreas, constituting an important receptor subfamily that is instrumental in the progression and metastasis of cancer 38, 39.

Integrin αvβ8 is far less studied in cancers than other members of the integrin αv-subfamily. It has been confirmed that the tumor cell is the main compartment where αvβ8 is expressed 19. When compared to hematogenous- and lymphoid-derived malignant lines, αvβ8 is significantly enriched in carcinoma, glioma, and melanoma 21, 40. The current study provides strong evidence that integrin αvβ8 may be expressed in colon cancer, as the expression rate in resected samples was 36.9%. For most human colon cancer cells, high expression of integrin αvβ8 was detected. Additionally, our results show that αvβ8 expression is significantly associated with lymph node metastasis, distant metastasis of tumors, and clinical TNM stage. According to the Cox proportional hazard model and survival analysis, we have shown that integrin αvβ8 predicts a poor prognosis for colon cancer patients. Therefore, in addition to being a potential immune-histochemical marker for lymph node metastasis and distant metastasis, integrin αvβ8 staining in surgical specimens could serve as a clinical prognostic marker of colon cancer.

Studies showed that integrin αvβ8, which was highly expressed on the tumor cell surface but not on immune cells, inhibited CD8^+^TIL response and the recruitment of immune cells to tumor centers 19. CD8^+^TILs are crucial components of the tumor- specific cellular adaptive immunity that attacks tumor cells. In colon cancer, density and location of CD8^+^TILs have a prognostic value superior to TNM classification 41. Here, we demonstrated that integrin αvβ8 may be involved in the immunosuppression of colon cancer by prohibiting the invasion of CD8^+^TILs. Our results indicate that αvβ8 may not only significantly promote progression and metastasis of colon cancer but also evade host immunity; therefore, integrin αvβ8 may represent a potential therapeutic target in the treatment of colon cancer. Thus, it is essential that we gain a complete understanding of the role that the interaction between integrin αvβ8, colon cancer cells, and its immediate environment plays in colon cancer. While αvβ8 has been shown to promote motility and TGF-β1 activation in a number of tumor types, no study has yet examined how these functions are linked, especially in colon cancer.

Besides the effects of integrin bidirectional signaling on tumors, it has been reported that RGD-binding integrins are also the main regulators of TGF-β which could become tumor-promoting by acting directly on the tumor cells to drive invasion and indirectly by promoting a tumor-permissive microenvironment 16, 31. Some kinds of these integrins have been strongly implicated in the promotion of tumor growth and metastasis of many different types of cancer by the activation of TGF-β 17. Integrin αvβ8 binds to LAP and brings the latent complexes into proximity with the membrane-bound protease that cleaves LAP. Here, we revealed that integrin αvβ8-expressing colon cancer cells, SW620 and HT-29, were able to bind to LAP; moreover, that this binding was significantly inhibited by αvβ8 antibodies or β8-siRNA. Additionally, we explored whether αvβ8 on colon cancer cells mediated TGF-β1 activation. Similarly, the TGF-β1 activation of colon cancer was greatly reduced when treated with αvβ8 antibodies or β8-siRNA, which indicated that integrin αvβ8 was a mediator of latent TGF-β1 activation for colon cancer. Effects of integrin-depended TGF-β activation on tumor cells are performed in a "paracrine" way 42. Thus, integrin on one tumor cell presents active TGF-β which then bind to TGF-β receptors (TGFβR) on adjacent cells, enhancing migration and invasiveness during tumor progression. As a result, we hypothesize that by interacting with LAP, integrin αvβ8 expressed on colon cancer cells activates TGF-β1 secreted by cancer cells, and then active TGF-β1 acts on adjacent cells, which likely results in driving invasion and metastasis of colon cancer.

The integrin αvβ8-TGF-β1 axis contributes to the regulation of angiogenesis and invasiveness of glioblastoma 40. Altering integrin αvβ8 levels had an obvious effect on the angiogenic and invasive growth properties of glioblastoma, in part, reflected by a diminished activation of latent TGF-β 43. However, the role of integrin αvβ8 in invasion and migration of epithelial neoplasms, especially colon cancer has not been well elucidated. Here, we examined the effect of integrin αvβ8 on the TGF-β1 induced migration and invasion of colon cancer cells. Our data demonstrated that αvβ8 promoted the migration and invasion of colon cancer cells by activating TGF-β1, as both αvβ8 antibodies and β8-siRNA could abolish the effect of latent TGF-β1. These results indicate that integrin αvβ8 on colon cancer is an activator of TGF-β1, which contributes to the invasion and migration of colon cancer cells. Additionally, our previous research demonstrated that integrin αvβ6 could increase the expression and secretion of MMP-9 in colon cancer and cholangiocarcinoma, which also plays a role in migration of these two cancers 44. Recently, metastasis of cancer cells has been shown to be associated with TGF-β1-mediated upregulation of MMP-9^45^. We analyzed the effects of integrin αvβ8-induced TGF-β1 activation on expression and activity of MMP-9 in colon cancer. Integrin αvβ8 was found to not only increase the expression of MMP-9 but also promote its activity in colon cancer cells under the condition of latent TGF-β1. Our previous data demonstrated that there was a direct link between integrin αvβ6 and ERK2, which activates ETS transcription factors Ets-1, leading to the upregulation of MMP-9^46^. In comparison with integrin αvβ6, integrin αvβ8 upregulated MMP-9 through activation of TGF-β1. However, whether there is an “outside in” signaling of integrin αvβ8 in colon cancer and its role in cancer migration and invasion needs to be further explored.

Colon cancer still has a poor prognosis because of its inclination for distant metastasis. In the present study, we present a novel marker, integrin αvβ8, as an unfavorable indicator in colon cancer. Moreover, we confirmed that integrin αvβ8 on colon cancer cells activates TGF-β1 by binding to LAP, which has a large contribution towards migration and invasion. This finding advances our understanding of the mechanisms of colon cancer progression; it may lead to the development of integrin αvβ8-based strategies for effective therapeutic approaches in colon cancer.

## Figures and Tables

**Table 1:** Relation between integrin αvβ8 expression and clinicopathologic variables in colon cancer cases

Clinicopathological factors	n (165)	Αvβ8 expression		P Value
		Positive (n=61)	Negative (n=104)	
**Gender**				0.227
Male	90	37	53	
Female	75	24	51	
**Age(years)**				0.072
≤60	85	37	48	
>60	80	24	56	
**Tumor anatomical location**				0.787
Right hemicolon cancer	87	33	54	
Left hemicolon cancer	78	28	50	
**T stage**				0.086
T1#	4	1	3	
T2#	19	5	14	
T3#	66	19	47	
T4#	76	36	40	
**N stage**				0.007
N0	93	26	67	
N1	43	24	19	
N2	29	11	18	
**M stage**				0.029
M0	124	40	84	
M1	41	21	20	
**TNM stage**				0.018
I - II	82	23	59	
III-IV	83	38	45	
**Differentiation**				<0.001
Well	67	19	48	
Moderate	59	10	49	
Poor/undifferentiated	39	32	7	
**CD8+TILs density**				<0.001
Low	73	38	35	
high	92	23	69	
**Survival (60-month follow-up)**				0.003
Death	78	38	40	
Censored	87	23	64	

^*^Log-rank test

**Table 2:** Univariate and multivariate analysis of association of clinicopathologic features with 5-year survival in colon cancer

Variable	Univariate analysis				Multivariate analysis		
	Relative risk	95% CI	P Value		Relative risk	95% CI	P Value
**Age at diagnosis**	1.063	1.037, 1.068	0.026		1.085	1.042, 1.082	0.007
**Gender**							
Male	1.000(Ref.)						
Female	0.847	0.487, 1.212	0.535				
**Tumor anatomical location**							
Right hemicolon cancer	1.000(Ref.)						
Left hemicolon cancer	0.653	0.515, 1.164	0.361				
**T stage**							
T1	0.021	0.000, > 10^5^	0.892				
T2	0.561	0.756, 3.251	0.294				
T3	0.975	0.597, 1.579	0.977				
T4	1.000(Ref.)						
**N stage**							
N0	1.000(Ref.)						
N1	1.218	0.627, 2.420	0.534				
N2	1.527	0.875, 3.087	0.135				
**M stage**							
M0	1.000(Ref.)				1.000(Ref.)		
M1	1.531	0.912, 3.142	0.029		1.854	0.967, 3.238	0.037
**TNM stage**							
I	1.000(Ref.)						
II	0.573	0.256, 1.387	0.235				
III	0.674	0.295, 1.765	0.474				
IV	1.264	0.445, 2.789	0.847				
**Differentiation**							
Well	1.000(Ref.)				1.000(Ref.)		
Moderate	0.901	0.567, 1.723	0.723		1.327	0.573, 1.658	0.256
poor/undifferentiated	2.362	1.406, 4.621	0.002		1.653	0.785, 2.973	0.056
**αvβ8**							
Negative	1.000(Ref.)				1.000(Ref.)		
Positive	2.224	1.280, 3.675	<0.001		1.681	0.976, 3.109	0.031

## Abbreviations

ECM: extracellular matrix
ELISA: enzyme- linked immunosorbent assay
ERK2: extracellular signal-regulated kinase-2
HE: hematoxylin-eosin
LAP: latency-associated peptide
MMP: matrix metalloproteinases
PBS: phosphate buffered saline
RGD: arginine-glycine-aspartic acid
SDS-PAGE: sodium dodecyl sulfate-polyacrylamide gel electrophoresis
SD: standard deviation
TIL: tumor-infiltrating lymphocytes
TMLC: transformed mink lung epithelial cells
TNM: tumor-node-metastasis

## References

[1] Bray F, Ferlay J, Soerjomataram I, Siegel RL, Torre LA, Jemal A (2018). Global cancer statistics 2018: GLOBOCAN estimates of incidence and mortality worldwide for 36 cancers in 185 countries. CA Cancer J Clin.

[2] Kopetz S, Chang GJ, Overman MJ, Eng C, Sargent DJ, Larson DW (2009). Improved survival in metastatic colorectal cancer is associated with adoption of hepatic resection and improved chemotherapy. J Clin Oncol.

[3] Liang B, Li L, Miao R, Wang J, Chen Y, Li Z (2019). Expression of Interleukin-6 and Integrin ανβ6 in Colon Cancer: Association with Clinical Outcomes and Prognostic Implications. Cancer Invest.

[4] Hynes RO (2002). Integrins: bidirectional, allosteric signaling machines. Cell.

[5] Zhu J, Zhu J, Springer TA (2013). Complete integrin headpiece opening in eight steps. J Cell Biol.

[6] Huveneers S, Danen EH (2009). Adhesion signaling - crosstalk between integrins, Src and Rho. J Cell Sci.

[7] Harburger DS, Calderwood DA (2009). Integrin signalling at a glance. J Cell Sci.

[8] Schittenhelm J, Klein A, Tatagiba MS, Meyermann R, Fend F, Goodman SL (2013). Comparing the expression of integrins αvβ3, αvβ5, αvβ6, αvβ8, fibronectin and fibrinogen in human brain metastases and their corresponding primary tumors. Int J Clin Exp Pathol.

[9] Niu J, Li Z (2017). The roles of integrin αvβ6 in cancer. Cancer Lett.

[10] Jin H, Varner J (2004). Integrins: roles in cancer development and as treatment targets. Br J Cancer.

[11] Desgrosellier JS, Cheresh DA (2010). Integrins in cancer: biological implications and therapeutic opportunities. Nat Rev Cancer.

[12] Sheldrake HM, Patterson LH (2014). Strategies to inhibit tumor associated integrin receptors: rationale for dual and multi-antagonists. J Med Chem.

[13] Nishimura SL, Sheppard D, Pytela R (1994). Integrin alpha v beta 8. Interaction with vitronectin and functional divergence of the beta 8 cytoplasmic domain. J Biol Chem.

[14] Ozawa A, Sato Y, Imabayashi T, Uemura T, Takagi J, Sekiguchi K (2016). Molecular Basis of the Ligand Binding Specificity of αvβ8 Integrin. J Biol Chem.

[15] Worthington JJ, Klementowicz JE, Travis MA (2011). TGFβ: a sleeping giant awoken by integrins. Trends Biochem Sci.

[16] Brown NF, Marshall JF (2019). Integrin-Mediated TGFβ Activation Modulates the Tumour Microenvironment. Cancers (Basel).

[17] Nieberler M, Reuning U, Reichart F, Notni J, Wester HJ, Schwaiger M (2017). Exploring the Role of RGD-Recognizing Integrins in Cancer. Cancers (Basel).

[18] Mu D, Cambier S, Fjellbirkeland L, Baron JL, Munger JS, Kawakatsu H (2002). The integrin alpha(v)beta8 mediates epithelial homeostasis through MT1-MMP-dependent activation of TGF-beta1. J Cell Biol.

[19] Takasaka N, Seed RI, Cormier A, Bondesson AJ, Lou J, Elattma A (2018). Integrin αvβ8-expressing tumor cells evade host immunity by regulating TGF-β activation in immune cells. JCI Insight.

[20] Mertens-Walker I, Fernandini BC, Maharaj MS, Rockstroh A, Nelson CC, Herington AC (2015). The tumour-promoting receptor tyrosine kinase, EphB4, regulates expression of integrin-β8 in prostate cancer cells. BMC Cancer.

[21] He J, Liu Y, Zhang L, Zhang H (2018). Integrin Subunit beta 8 (ITGB8) Upregulation Is an Independent Predictor of Unfavorable Survival of High-Grade Serous Ovarian Carcinoma Patients. Med Sci Monit.

[22] Stockis J, Liénart S, Colau D, Collignon A, Nishimura SL, Sheppard D (2017). Blocking immunosuppression by human Tregs in vivo with antibodies targeting integrin αVβ8. Proc Natl Acad Sci U S A.

[23] Edwards JP, Thornton AM, Shevach EM (2014). Release of active TGF-β1 from the latent TGF-β1/GARP complex on T regulatory cells is mediated by integrin β8. J Immunol.

[24] Hayashido Y, Kitano H, Sakaue T, Fujii T, Suematsu M, Sakurai S (2014). Overexpression of integrin αv facilitates proliferation and invasion of oral squamous cell carcinoma cells via MEK/ERK signaling pathway that is activated by interaction of integrin αvβ8 with type Ⅰ collagen. Int J Oncol.

[25] Ahmed N, Niu J, Dorahy DJ, Gu X, Andrews S, Meldrum CJ (2002). Direct integrin alphavbeta6-ERK binding: implications for tumour growth. Oncogene.

[26] Niu J, Dorahy DJ, Gu X, Scott RJ, Draganic B, Ahmed N (2002). Integrin expression in colon cancer cells is regulated by the cytoplasmic domain of the beta6 integrin subunit. Int J Cancer.

[27] Liang B, Shahbaz M, Wang Y, Gao H, Fang R, Niu Z (2015). Integrinβ6-targeted immunoliposomes mediate tumor-specific drug delivery and enhance therapeutic efficacy in colon carcinoma. Clin Cancer Res.

[28] Abe M, Harpel JG, Metz CN, Nunes I, Loskutoff DJ, Rifkin DB (1994). An assay for transforming growth factor-beta using cells transfected with a plasminogen activator inhibitor-1 promoter-luciferase construct. Anal Biochem.

[29] Li J, Wang J, Chen R, Bai Y, Lu X (2017). The prognostic value of tumor-infiltrating T lymphocytes in ovarian cancer. Oncotarget.

[30] Teng MW, Ngiow SF, Ribas A, Smyth MJ (2015). Classifying Cancers Based on T-cell Infiltration and PD-L1. Cancer Res.

[31] Khan Z, Marshall JF (2016). The role of integrins in TGFβ activation in the tumour stroma. Cell Tissue Res.

[32] Villalba M, Evans SR, Vidal-Vanaclocha F, Calvo A (2017). Role of TGF-β in metastatic colon cancer: it is finally time for targeted therapy. Cell Tissue Res.

[33] Sun L, Diamond ME, Ottaviano AJ, Joseph MJ, Ananthanarayan V, Munshi HG (2008). Transforming growth factor-beta 1 promotes matrix metalloproteinase-9- mediated oral cancer invasion through snail expression. Mol Cancer Res.

[34] Sinpitaksakul SN, Pimkhaokham A, Sanchavanakit N, Pavasant P (2008). TGF-beta1 induced MMP-9 expression in HNSCC cell lines via Smad/MLCK pathway. Biochem Biophys Res Commun.

[35] Paduch R, Kandefer-Szerszeń M, Szuster-Ciesielska A, Plewka K (2010). Transforming growth factor-beta1 modulates metalloproteinase-2 and -9, nitric oxide, RhoA and alpha-smooth muscle actin expression in colon adenocarcinoma cells. Cell Biol Int.

[36] Horwitz AR (2012). The origins of the molecular era of adhesion research. Nat Rev Mol Cell Biol.

[37] Kechagia JZ, Ivaska J, Roca-Cusachs P (2019). Integrins as biomechanical sensors of the microenvironment. Nat Rev Mol Cell Biol.

[38] Li ZH, Zhou Y, Ding YX, Guo QL, Zhao L (2019). Roles of integrin in tumor development and the target inhibitors. Chin J Nat Med.

[39] Bandyopadhyay A, Raghavan S (2009). Defining the role of integrin alphavbeta6 in cancer. Curr Drug Targets.

[40] Tchaicha JH, Reyes SB, Shin J, Hossain MG, Lang FF, McCarty JH (2011). Glioblastoma angiogenesis and tumor cell invasiveness are differentially regulated by β8 integrin. Cancer Res.

[41] Pagès F, Kirilovsky A, Mlecnik B, Asslaber M, Tosolini M, Bindea G (2009). In situ cytotoxic and memory T cells predict outcome in patients with early-stage colorectal cancer. J Clin Oncol.

[42] Sheppard D (2005). Integrin-mediated activation of latent transforming growth factor beta. Cancer Metastasis Rev.

[43] Guerrero PA, Tchaicha JH, Chen Z, Morales JE, McCarty N, Wang Q (2017). Glioblastoma stem cells exploit the αvβ8 integrin-TGFβ1 signaling axis to drive tumor initiation and progression. Oncogene.

[44] Gu X, Niu J, Dorahy DJ, Scott R, Agrez MV (2002). Integrin alpha(v)beta6-associated ERK2 mediates MMP-9 secretion in colon cancer cells. Br J Cancer.

[45] Joseph MJ, Dangi-Garimella S, Shields MA, Diamond ME, Sun L, Koblinski JE (2009). Slug is a downstream mediator of transforming growth factor-beta1-induced matrix metalloproteinase-9 expression and invasion of oral cancer cells. J Cell Biochem.

[46] Gao H, Peng C, Liang B, Shahbaz M, Liu S, Wang B (2014). β6 integrin induces the expression of metalloproteinase-3 and metalloproteinase-9 in colon cancer cells via ERK-ETS1 pathway. Cancer Lett.

